# Supplementary material for: The Evolution and Characterization of the RNA Interference Pathways in Lophotrochozoa
Source: Genome Biol Evol. 2024 May 7;16(5):evae098. doi: 10.1093/gbe/evae098 (PMC11114477; doi:10.1093/gbe/evae098)
Supplement: evae098_Supplementary_Data [file evae098_supplementary_data.zip › Supplementary_materials.zip!/selected_miRNAs/NoGe_selected_miRNAs/mirdeep_pdf_files/NMRB01001012.1_Notospermus_geniculatus_strain_Ushimado_scaffold1012,_whole_genome_shotgun_sequence_23918.pdf]

Provisional ID	:	NMRB01001012.1_Notospermus_geniculatus_strain_Ushimado_scaffold1012,_whole_genome_shotgun_sequence_23918
Score total	:	481.9
Score for star read(s)	:	3.9
Score for read counts	:	477.3
Score for mfe	:	1.3
Score for randfold	:	0
Score for cons. seed	:	-0.6
Total read count	:	948
Mature read count	:	921
Loop read count	:	0
Star read count	:	27

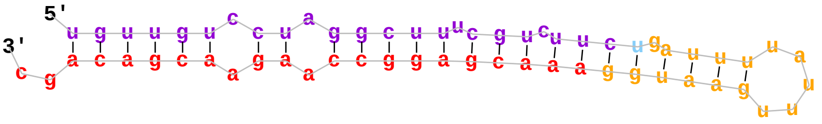

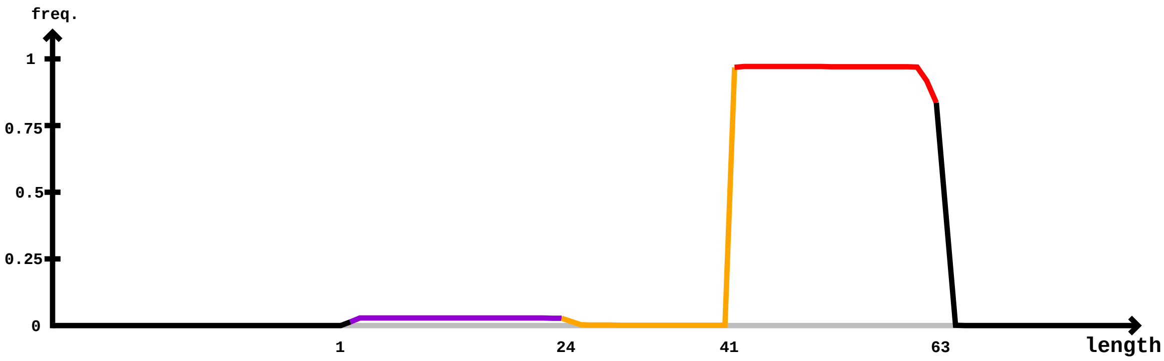


```
                                   Star                                    Mature
5'- accuucuggauacaacaagcuaguacgacuguuguccuaggcuuucgucuucugauuuuauuugaauggaaacgaggccaagaacgacagcugcacuggcaugcgucaucg   -3'   obs
    accuucuggauacaacaagcuaguacgacuguuguccuaggcuuucgucuucugauuuuauuugaauggaaacgaggccaagaacgacagcugcacuggcaugcgucaucg         exp
    ........(((((..((.((((((.((.(((((((.((.(((((.(((.((((.((((.....)))))))))))))))).)).))))))).)).)))))).)).)).))).   reads   mm   sample
    .....................................................................aaacgaggccaagaacgacaC.....................   1       1    Fm2
    .....................................................................aaacgaggccaagaacgacagU....................   52      1    Fm2
    .....................................................................aaacgaggccaagaacgacaCc....................   9       1    Fm2
    .....................................................................aaacgaggccaagaacgacagUu...................   9       1    Fm2

    .............................uguuguccuaggcuuucgucuuc...........................................................   8       0    Fm1
    ..............................guuguccuaggcuuucgucG.............................................................   1       1    Fm1
    ..............................guuguccuaggcuuucgucuuc...........................................................   6       0    Fm1
    ..............................guuguccuaggcuuucgucuucu..........................................................   2       0    Fm1
    ..............................guuguccuaggcuuucgCcuucu..........................................................   1       1    Fm1
    .....................................................................aaacgaggccaagaacgacag.....................   6       0    Fm1
    .....................................................................aaacgaggccaagaacgacaC.....................   1       1    Fm1
    .....................................................................aaacgaggccaagaacgacagU....................   99      1    Fm1
    .....................................................................aaacgaggccaagaacgacagc....................   60      0    Fm1
    .....................................................................aaacgaggccaagaacgacagA....................   8       1    Fm1
    .....................................................................aaacgaggccaagaacgacaCc....................   2       1    Fm1
    .....................................................................aaacgaggccaagaacgacagUu...................   3       1    Fm1
    .....................................................................aaacgaggccaagaacgacagcA...................   8       1    Fm1
    ......................................................................aacgaggccaagaacgacagc....................   1       0    Fm1

    .....................................................................aaacgaggccaagaacgacaC.....................   2       1    Mm1
    .....................................................................aGacgaggccaagaacgacagc....................   1       1    Mm1
    .....................................................................aaacgaggccaagaacgacaCc....................   10      1    Mm1
    .....................................................................aaacgaggccaagaacgacagU....................   7       1    Mm1
    .....................................................................aaacgaggccaagaacgacagA....................   16      1    Mm1
    .....................................................................aaacgaggccaagaacgacagc....................   5       0    Mm1

    .............................uguuguccuaggcuuucgucuuc...........................................................   5       0    Mm2
    ..............................guuguccuaggcuuucgucuuc...........................................................   1       0    Mm2
    ..............................guuguccuaggcuuucgucuucU..........................................................   1       1    Mm2
    ....................................................ugauuuuauuugaauggaaacgaggcc................................   1       0    Mm2
    .....................................................................aaacgaggccaagaacgaca......................   5       0    Mm2
    .....................................................................aaacgaggccaagaacgacaU.....................   1       1    Mm2
```

Star **Mature**

```
accuucuggauacaacaagcuaguacgacuguuguccuaggcuuucgucuucugauuuuauuugaauggaaacgaggccaagaacgacagcugcacuggcaugcgucaucg
.....................................................................aaacgaggccaagaacgacaC.....................   1     1     Mm2
.....................................................................aaacgaggccaagaacgacag.....................   3     0     Mm2
.....................................................................aaacgaggccaagaacgacaCc....................   6     1     Mm2
.....................................................................aaacgaggccaagaacgacagU....................   108   1     Mm2
.....................................................................aaacgaggccaagaacgacagA....................   4     1     Mm2
.....................................................................aaacgaggccaagaacgacagc....................   34    0     Mm2
.....................................................................aaacgaggccaagaacgUcagc....................   1     1     Mm2
.....................................................................aaacgaggccaagaacgacagUu...................   3     1     Mm2
.....................................................................aaacgaggccaagaacgacaCcu...................   3     1     Mm2
.....................................................................aaacgaggccaagaacgacagcA...................   3     1     Mm2

.....................................................................aaacgaggccaagaacgac.......................   1     0     Fm3
.....................................................................aaacgaggccaagaacgaca......................   38    0     Fm3
.....................................................................aaacgaggccaagaacCaca......................   1     1     Fm3
.....................................................................aaacgaggccaagaacgacag.....................   41    0     Fm3
.....................................................................aaacgaggccaagaacgacaC.....................   9     1     Fm3
.....................................................................aaacgaggccaagaacCacag.....................   1     1     Fm3
.....................................................................aaacgaggccaagaacgacagU....................   141   1     Fm3
.....................................................................aaacgaggccaagaacgacagc....................   65    0     Fm3
.....................................................................aaacgaggccaagaacgacaCc....................   9     1     Fm3
.....................................................................aaacgaggccaagaacgGcagc....................   1     1     Fm3
.....................................................................aaacgaggccaagaacgacagA....................   15    1     Fm3
.....................................................................aaacgaggcUaagaacgacagc....................   1     1     Fm3
.....................................................................aaacgaggccaagaacgCcagc....................   1     1     Fm3
.....................................................................aaacgaggccaagaacgacagcu...................   1     0     Fm3
.....................................................................aaacgaggccaagaacgacagcA...................   3     1     Fm3
.....................................................................aaacgaggccaagaacgacagUu...................   3     1     Fm3
.....................................................................aaacgaggccaagaacgacagcuU..................   1     1     Fm3
......................................................................aacgaggccaagaacgacagU....................   1     1     Fm3

..............................guuguccuaggcuuucgucuucu..........................................................   1     0     Mm3
..............................guuguccuaggcuuucgucuucugauu......................................................   1     0     Mm3
.....................................................................aaacgaggccaagaacgaca......................   5     0     Mm3
.....................................................................aaacgaggccaagaacgacag.....................   11    0     Mm3
.....................................................................aaacgaggccaagaacgCcag.....................   1     1     Mm3
.....................................................................aaacgaggccaagaacgacagc....................   50    0     Mm3
.....................................................................aaacgaggccaagaacgacaCc....................   4     1     Mm3
.....................................................................aaacgaggccaagaacgacagU....................   40    1     Mm3
.....................................................................aaacgaggccaagaacgacagA....................   2     1     Mm3
.....................................................................aaacgaggccaagaacgacagUu...................   1     1     Mm3
.....................................................................aaacgaggccaagaacgacagcA...................   1     1     Mm3
......................................................................aacgaggccaagaacgacagc....................   1     0     Mm3
```
